# Reply: SUN *vs* BEV*þ*IFN in first-line mRCC therapy: no evidence for a statistically significant difference in progression-free survival

**DOI:** 10.1038/sj.bjc.6605447

**Published:** 2010-01-05

**Authors:** J S Thompson Coon, Z Liu, M Hoyle, G Rogers, C Green, T Moxham, K Welch, K Stein

**Affiliations:** 1 Peninsula Technology Assessment Group, Peninsula Medical School, University of Exeter, Noy Scott House, Barrack Road, Exeter EX2 5DW, UK; 2 Wessex Institute, University of Southampton, Mailpoint 728, Boldrewood, Southampton SO16 7PX, UK

**Sir,**

We thank you for providing us the opportunity to respond to the Letter to the Editor received by your office in response to our article ‘Sunitinib and bevacizumab for first-line treatment of metastatic renal cell carcinoma: a systematic review and indirect comparison of clinical effectiveness’ (Thompson Coon *et al*, 2009). We are pleased that Professor Mickisch recognises the need for and importance of indirect treatment comparisons in the absence of head-to-head comparisons and welcome a debate on the relative merits of indirect treatment comparison methods.

It appears that some of the comments outlined by Professor Mickisch stem from a misunderstanding of the methods used in our analysis. Ideally we would have given a more detailed account in the paper, but owing to restrictions in the length of the article we chose to rely on the cited reference (Ades, 2003) to provide a fuller description of the methods used. Our analysis was mainly conducted within the Bayesian framework and as such issues relating to frequentist hypothesis testing are not applicable.

Our analysis compared the reported differences (hazard ratios) between the three treatments rather than comparing the absolute effects of sunitinib with those of bevacizumab plus IFN and those of IFN alone. Accordingly, it is important to clarify that we did not use a one-sided *t*-test to calculate the *P*-value, as inferred by Professor Mickisch. We acknowledge that the language used in describing the output from the MCMC as a one-sided *P*-value may give rise to some interpretation issues for an audience who are more familiar with frequentist hypothesis testing than Bayesian analysis. The *P*-value obtained from the MCMC – although regarded as equivalent to that obtained from a one-sided test – is in reality a direct estimate of the probability that one treatment is better than another. It is one of the advantages of the Bayesian approach that it enables intuitive probability statements to be made about propositions; in this case, 0.0272 is an estimate of the probability that bevacizumab provides superior PFS gain to sunitinib. As a result, within the context of our analysis, it would have been neither possible nor desirable to adopt the methods suggested by Professor Mickisch, which relate solely to the frequentist paradigm.

In terms of the number of simulations used in our analysis, we are unaware of any references suggesting that simulation numbers should be as low as 2000 iterations in an MCMC and we would be interested in the source of Professor Mickisch's comments. It is important to clarify that we were not simulating individual patient experiences but rather repeatedly sampling the differences between treatments in each of the uncertain distributions (i.e., our analysis was at the level of a cohort of patients as reflected in trial results). Accordingly, cautions that apply to, for example, bootstrap sampling of individual patient data are not applicable in this instance. Increasing the number of simulations in an MCMC will produce a more accurate estimate (with less possibility of the outputs being distorted by outlying or extreme simulations), but will not result in spurious certainty (in other words, the posterior distribution will become smoother, but not narrower).

Notwithstanding the relevance of guidance from the Canadian Agency for Drugs and Technologies in Health (CADTH) to this work, we do not share Professor Mickisch's conclusion that they have identified the Bucher method as the ‘gold standard’ method for performing indirect treatment comparisons. The CADTH identifies both the Bucher and the Bayesian multiple treatment comparison MTC methods as suitable for conducting indirect treatment comparisons, and concludes that the MTC is elegant and more widely applicable, although it may be perceived to be computationally complex. We feel that the MTC approach is more appropriate in this instance as it uses the data from all three available studies in one analysis, thereby handling all the uncertainty at the same time. In contrast, the Bucher method necessitates a two-step analysis in which we would first need to meta-analyse the results from the two trials of bevacizumab plus IFN *vs* IFN and then perform the indirect comparison of this pooled result with the result of the trial of sunitinib *vs* IFN. In fact, because the number of trials is small and the evidence network simple, in this instance, performing this analysis on the dataset used in our article produces results that are almost identical to those generated in the Bayesian analysis: HR=0.796 (95% CI: 0.629, 1.007).

As our analysis was concerned with differences between treatments estimated in randomised comparisons, we were unable to utilise the data originating from the single-arm expanded access trial (Gore *et al*, 2007) cited in the letter, in which participants received sunitinib only. We are unclear as to why Professor Mickisch does not regard the trial by Rini and colleagues as providing useful data to incorporate into his ongoing research. Although the trial was open-label in design, a large number of individuals (*n*=732) were randomised to receive either bevacizumab plus IFN or IFN alone, and the treatment effects reported to date with respect to progression-free survival are similar to those seen in the trial reported by Escudier and colleagues. We assume that a full peer-reviewed presentation of Professor Mikisch's methods, data, and results will be available in due course, and look forward to reading his findings.

We hope that these points serve to clarify both the methods and the outputs of our analysis and once again thank you for giving us the opportunity to engage in the debate.
